# Anatomical Covariance Analysis: Detection of Disrupted Correlation Network Related to Clinical Trait Fatigue in Multiple Sclerosis: A Pilot Study

**DOI:** 10.1155/2020/5807496

**Published:** 2020-12-23

**Authors:** Rosalia Dacosta-Aguayo, Glenn Wylie, John DeLuca, Helen Genova

**Affiliations:** ^1^Neuropsychology and Neuroscience, Kessler Foundation, 120 Eagle Rock Avenue, Suite 100, East Hanover, New Jersey 07936, USA; ^2^Department of Physical Medicine and Rehabilitation, Rutgers University, New Jersey Medical School, Newark, NJ 07101, USA; ^3^Rocco Ortenzio Neuroimaging Center, Kessler Foundation, West Orange, NJ, USA

## Abstract

**Background:**

Fatigue is one of the most distressing symptoms among persons with multiple sclerosis (PwMS). The experience of fatigue is inherently interoceptive, yet no study to date has explicitly investigated the insular cortex (IC) as a primary goal in the experience of fatigue in PwMS. In addition, it is unknown how brain regions such as IC play a role in state or trait fatigue.

**Objective:**

Assess the involvement of the IC in trait fatigue and state fatigue in PwMS with and without clinical fatigue.

**Methods:**

Trait and state fatigue, cognitive status, and structural MRI were assessed in 27 PwMS. PwMS were stratified into nonclinical fatigue (nF-MS, FSS ≤ 4.0) (*n* = 10) and clinical fatigue (F-MS, FSS ≥ 5.0) (*n* = 10). Voxel-based morphometry analysis (VBM) for the whole sample (*n* = 20) and for the two groups was performed. Anatomical covariance analysis (ACA) analysis was conducted by selecting different volumes included in the corticostriatal network (CoStN) and analyzing interhemispheric correlations between those volumes to explore the state of the CoStN in both groups.

**Results:**

In the VBM analysis, when considering the whole sample of PwMS, higher levels of trait fatigue were negatively associated with grey matter (GM) volume in the left dorsal anterior insula (dAI) (rho = −0.647; *p* = 0.002; *R*^2^ = 0.369). When comparing nF-MS versus F-MS, significant differences were found in the left dAI, where the F-MS group showed less GM volume in the left dAI. In the ACA analysis, the F-MS group showed fewer significant interhemispheric correlations in comparison with the Low-FSS group.

**Conclusions:**

The present results provide support to the interoceptive component of self-reported fatigue and suggest that changes in the relationship between the different anatomical regions involved in the CoStN are present even in nonclinical trait fatigue. Those changes might be responsible for the experience of trait fatigue in PwMS. Future studies with larger samples and multimodal MRI acquisitions should be considered to fully understand the changes in the CoStN and the specific role of the IC in trait fatigue.

## 1. Introduction

Fatigue is one of the most distressing symptoms among persons with multiple sclerosis (PwMS), with an estimated prevalence between 52%-98% [1]. Defined as “An overwhelming sense of tiredness, lack of energy and a feeling of exhaustion” [2], fatigue has a negative impact on activities of daily living [3].

Despite the high prevalence and impact of fatigue in PwMS, the neuronal correlates involved in its development remain poorly understood. Whereas there is no generally accepted nomenclature that clearly defines fatigue, two different forms of fatigue have been proposed: primary and secondary fatigue [4]. Primary fatigue has been proposed as the combination of immune-related neuroinflammation processes associated with the disease itself, such as the immune system dysfunction [5], axonal demyelination [6], white matter disruption [7], and grey matter (GM) atrophy [8]. Conversely, secondary fatigue is caused by secondary factors such as poor sleep and/or excessive daytime sleepiness, physical deconditioning, and depression [9], which, although coexist with primary fatigue, are not considered the primary source of fatigue [10]. In addition to the distinction made between primary and secondary fatigue, some have argued that fatigue can be divided into two descriptive forms: state fatigue (i.e., the feeling of fatigue in the moment) and trait fatigue (i.e., the feeling of fatigue over an extended period) [11]. State fatigue has been studied primarily with task-based functional magnetic resonance imaging (fMRI) studies in which attentional resources are required and sustained during a long period of time. Therefore, both the difficulty/cognitive loads of the task and the time on task are key components to assess how state fatigue increases across time. Those two components can only be assessed with fMRI paradigms in which fluctuations of functional activity can be related to changes [12] in state fatigue. Thus, we do not expect to find structural MRI correlates with state fatigue.

Structural and functional neuroimaging studies that have focused on the study of neural correlates of fatigue suggesting the role of the corticostriatal network (CoStN) in fatigue, including brain regions such as the anterior cingulate cortex (ACC), thalamus, putamen, posterior cingulate cortex (PCC), caudate, and the amygdala. Additionally, most neuroimaging studies to date on fatigue in PwMS have focused primarily on the motor and the cognitive aspects of fatigue; there is a gap in the literature in relation to the interoceptive component of fatigue in PwMS. That is, the study of the subjective feeling of fatigue.

Recent work has hypothesized that the feeling of fatigue is related to a disruption of the interoceptive system [13, 14]. However, to date, only one study carried out by Gonzalez Campo et al., (2019) has tested this hypothesis empirically. In this study, the authors found decreased GM in the insular cortex (IC) in the clinically fatigued group (F-MS) when compared with a control group but not in the nonclinically fatigued group (nF-MS). Also, they found that IC volume was negatively associated with higher levels of fatigue in the F-MS but not in the nF-MS group. Additionally, they found that increased functional connectivity in interoceptive regions was also related to higher levels of fatigue, measured with the Modified Fatigue Impact Scale (MFIS). Nevertheless, they did not study how these interoceptive anatomical regions were related to other regions already described as being involved in fatigue in PwMS [15].

In the present work, we examined the interoceptive perception of fatigue severity, in particular, the IC, and how these interoceptive regions are related to the CoStN [16]. In this pilot study, three hypotheses were examined. First, we hypothesized that the IC will be involved in the perception of trait fatigue in the F-MS group but not in the nF-MS group. Second, we hypothesized that state fatigue will not be associated with structural damage in the IC. Third, we hypothesized that the F-MS group will show less GM correlations between different anatomical regions from the CoStN in comparison with the nF-MS group due to a disruption of the interoceptive system.

## 2. Methods

### 2.1. Participants

This is a reanalysis of existing data [17] in which a total of 27 PwMS [18] were first screened by phone to assess their medical and psychiatric history, problems and/or contraindications to perform an MRI scan, and their last exacerbation. Participants were excluded if they presented a significant history of neurological (other than MS) or psychiatric disorders, drug abuse, learning disabilities, current usage of steroids, benzodiazepines, or neuroleptics. Additionally, participants had to be relapse-free at least for 4 weeks [19]. The Institutional Review Board of Kessler Foundation approved the research protocol. All enrolled participants gave their written consent to participate in the study, and they were screened for MRI contraindications. The study was conducted following the ethical standards laid down in the 1964 Declaration of Helsinki and its following amendments.

### 2.2. Classification of the Participants

In a former study, Fatigue Severity Scale (FSS) was categorized into three different groups: [1] nonfatigue (FSS ≤ 4.0), [2] borderline fatigue (4.0 < FSS < 5.0), and [3] fatigue (FSS ≥ 5.0) [20]. In this study, the authors reported that comparing fatigue in patients at the extreme of a continuum (nonfatigue and fatigue) yielded significant correlations with cognitive capacity using the Minimal Assessment of Cognitive Function in MS (MACFIMS), whereas when including the borderline fatigue group in the analysis, the significance of the correlations was lost [20]. Furthermore, a criteria cut − off ≥ 5.0 was used in a recent publication to classify PwMS with pathological fatigue [21]. Therefore, we selected two groups (Low-FSS with ≤4.0 and High-FSS with ≥5.0) with the same number of participants in every group (*n* = 10) and discarded the 7 participants whose scoring fell into the borderline fatigue category (Table 1).

### 2.3. Procedure

PwMS were enrolled and screened for eligibility in the study providing written informed consent approved by the local Institutional Review Board. After providing consent, all subjects participated in an MRI scanning session (see below for scanning details). This protocol included both structural and functional scans. Structural scans were the ones reported in this study; the functional imaging results will be reported elsewhere.

On the day of the scan, participants' fatigue levels were assessed with the VAS_F [23] scale, which assesses fatigue symptoms experienced at the moment of the evaluation and was used as a measure of state fatigue. In the scale, participants are asked to report their level of fatigue on a scale from 0 to 100, with 0 being not fatigued and 100 being extremely fatigued. The Fatigue Severity Scale (FSS) [24], which focuses on fatigue symptoms experienced during the past week, was used as a measure of trait fatigue. The FSS is a widely used, reliable, precise, and clinical relevant tool, with validated cut-off [20, 21, 24] to assess fatigue severity [25, 26]. It is a 9-item scale in which participants are asked to choose a number from 1 to 7 that indicates the degree to which they agree with each of the statements, where 1 indicates strongly disagree and 7 indicates strongly agree. Higher scores on this scale indicate higher fatigue severity.

To assess general cognitive status, the oral response format from the Symbol Digit Modalities Test (SDMT) [27] was administered. Participants were asked to use a coded key to match nine abstracts symbols, which were paired with numerical digits. The final score was the number of correct matches in 90 seconds.

The FSS and the VAS_F were collected on the same day of the MRI scanning as a basal measure of the participants. Conversely, the SDMT was not collected on the same day of the MRI scanning (to avoid contamination by fatigue) but was collected within a week before the scan.

### 2.4. Scan Protocol

All MRI data were acquired on a Siemens Allegra 3.0T scanner equipped with a standard radio-frequency head coil at the medical school of the University of Medicine and Dentistry of New Jersey (now Rutgers University). The MRI protocol included one set of magnetization prepared rapid gradient echo (MPRAGE) T1-weighted images (Repetition Time (TR)/Echo Time (TE)/Flip Angle (FA): 2000 ms/4.38 ms/8°; Field of view (FOV) = 220 mm; in-plane resolution: 0.859 × 0.859 mm) and a fluid attenuation inversion recovery (FLAIR) image (TR/TE/FA: 8530 ms/81 ms/180°; FOV = 256 × 320 mm; in-plane resolution: 0.688 × 0.688 mm). All participants were screened for MRI contraindications.

### 2.5. Image Processing

#### 2.5.1. Brain Tissue Fraction

Total brain volume (TBV) for every individual subject was calculated from the T1-weighted images, using MRICloud software (https://mricloud.org/). Every segmented image was visually inspected. In case of doubt, a visual inspection from the technician in the MRICloud was requested. The advantage of using this software over other methods is that in MRICloud the anatomical scans are not registered to a single anatomical template. Instead, a Multiple-Atlas Likelihood Fusion (MALF) algorithm (Tang, et al., 2013) is used in which multiple atlases are considered during the registration. Total WM (TWM) and total (TGM) were calculated with the same software.

#### 2.5.2. Lesion Volume Analysis

A trained clinical neuropsychologist with knowledge in neurology and neuroanatomy and experience in lesion segmentation performed the segmentation of the lesions. Manual segmentation total lesion volume (TLV) was calculated in native space using ITK-SNAP (http://www.itksnap.org/pmwiki/pmwiki.php), an interactive medical imaging segmentation tool [28] by comparing T1 and FLAIR images. After this process, the mask was saved for every individual, and we used the following tools to convert the masks from native space to MNI space. First, we used the Linear Image Registration Tool (FLIRT) from FSL (http://www.fmrib.ox.ac.uk/fsl) to calculate the linear transformation matrix to register the T1 images to the MNI standard template. Second, we used the Nonlinear Image Registration Tool (FNIRT) from FSL to calculate the nonlinear transformation field. Third, we used the warp-fields created with FNIRT using the Applywarp tool from FSL. Then, we used fslmaths tool to add all the different lesions together for every single participant. We ensured that the masks were binarized after using all those transformations to avoid the problems with the edges in the masks. Finally, we calculated the whole volume load for every PwMS in standard space.

#### 2.5.3. Selection of the Regions of Interest

The left and right IC were defined anatomically by drawing them on the Montreal Neurological Institute (MNI) 152 standard brain. The IC's limits were taken considering the anterior, superior, and inferior periinsular sulci [29, 30]. The resulting left insula ROI fell within the following coordinates *x* = –23 and –43, *y* = –17 and 24, and *z* = –12 and 20; the right insula ROI fall within the reflection of those coordinates in the *x*-axis. The right and the left IC were divided into three regions by applying *k*-clustering algorithm to voxel-wise functional connectivity between the IC and the rest of the brain (see Deen et al., 2011 for a full description of the procedure) [31]. This was done bilaterally, resulting in six regions of interest (ROIs): dorsal anterior insula (dAI), ventral anterior insula (vAI), and posterior insula (pI) on both left and the right sides [29].

#### 2.5.4. Voxel-Based Morphometry Analysis

T1-weighted MRI images were analyzed with the FMRIB Software Library (FSL http://www.fmrib.ox.ac.uk/fsl), v6.0. Structural images were skull stripped and GM segmented. The resulting GM partial volume images were nonlinearly registered to the MNI152 standard template using FNIRT, which uses a b-spline representation of the registration warp field. The resulting images were averaged and flipped along the *x*-axis to create a left-right symmetric, study-specific GM template. Second, the native GM images nonlinearly registered to this study-specific template and “modulated” to correct for local expansion or contraction due to the nonlinear component of the spatial transformation by dividing them by the determinant of the Jacobian warp field. The modulated images were finally smoothed with an isotropic Gaussian kernel with a sigma of 3 mm. Finally, a general linear model (GLM) was applied to the six ROIs collapsed using 5000 permutation-based nonparametric testing, correcting for multiple comparisons across space only for the six ROIs collapsed. Age and sex were not introduced as covariates as they were not significantly different across both groups. First, we used the entire sample (*n* = 20) to assess the influence of FSS in GM for the six collapsed ROIs. Second, we divided the sample into nF-MS and F-MS groups to see if the results obtained for the whole sample were the same once was divided into nF-MS versus F-MS.

#### 2.5.5. Anatomical Covariance Analysis

A T_1_-multi-atlas segmentation function implemented in the MRICloud (https://mricloud.org/) [32] was applied to segment each T_1_-weighted image into a parcellation map including GM and WM structures [33]. The function provided by MRICloud is fully automated and fuses different algorithms (transformation algorithm, Large Deformation Diffeomorphic Metric Mapping (LDDMM), and the atlas label-fusion algorithm) [34–36] with a local search algorithm [37]. We used the atlas library version 10A, which includes 30 atlases from cognitively-normal individuals and individuals with cognitive impairment or dementia [33].

From the parcellation, we selected the following volumes of interest (VOIs) already reported to be involved in fatigue in PwMS [38–41]: right and left IC, anterior cingulate cortex (ACC), thalamus, putamen, posterior cingulate cortex (PCC), caudate, and the amygdala. The volumes considered were in native space, and they were normalized by dividing them by the TBV in native space. Cerebrospinal fluid was not considered.

For each group, we performed an anatomical covariance analysis (ACA) which consisted of the calculation of bivariate Pearson coefficients between the left and right pairs of each of the VOIs. This was done separately for the nF-MS and F-MS groups. We only considered interhemispheric correlations to avoid the problem of multiple comparisons with a small sample. The correlation coefficients were stored in an anatomical matrix of seven columns by seven rows. We used the Fisher's *Z* transformation of the correlation coefficients [42]. Finally, we used the Network-Based Statistic software (NBS) [43] (http://www.nitrc.org/projects/nbs/) to plot the significant correlations for the nF-MS and F-MS groups.

### 2.6. Statistical Analysis

For demographic, clinical, behavioral, and cognitive data, we used the Statistical Package for Social Sciences version 21.0 (SPSS Inc., Chicago, IL, USA). Normal distribution of data was tested with the Shapiro–Wilk test before each analysis. Group differences in demographic, cognitive, and behavioral characteristics were analyzed as follows: independent two-sample *t*-tests for normally distributed continuous variables; Mann–Whitney *U*-test for nonnormally distributed continuous variables; and Fisher's Exact test for categorical variables.

For the VBM analysis, we divided the sample (*n* = 20) into F-MS and nF-MS and we compared both groups. Results were family-wise error (FWE) corrected. Threshold-Free Cluster Enhancement (TFCE) was the method used to define the clusters [44].

For the volumetric analysis, a repeated 2 (GROUP: nF-MS versus F-MS) by 2 (IC VOLUME: left versus right), analysis of variance (ANOVA) was conducted to analyze the differences between the two groups in the right and left IC. Bivariate Pearson correlations were computed between FSS and VAS_F in the left and right IC volumes for the whole sample (*n* = 20) and for each group (nF-MS and F-MS) independently. Finally, bivariate interhemispheric correlations were conducted between the different VOIs as an exploratory analysis to explore the differences in the CoStN between the nF-MS and the F-MS groups.

## 3. Results

### 3.1. Demographical, Clinical, and Behavioral Data

There were no significant differences between the groups in age, gender, education, disease duration, MS type, state fatigue, or cognitive status. There was no significant correlation between the VAS_F and the FSS (*r* = 0.386; *p* = 0.093). By definition, the F-MS group had higher levels of trait fatigue than the nF-MS group (*t*_(14.9)_ = 13.6, *p* < 0.0001).

### 3.2. Brain Tissue Measurements

There were no significant differences between the nF-MS and the F-MS groups in TBV, (*F*_(1, 18)_ = 0.685, *p* = 0.419), total GM volume (GMV) (*F*_(1, 18)_ = 0.750, *p* = 0.398), total WM volume (WMV) (*F*_(1, 18)_ = 0.511, *p* = 0.484), and total lesion volume (*F*_(1, 18)_ = 0.954; *p* = 0.342) TLV). All values were expressed in milliliters (mL).

### 3.3. Voxel-Based Morphometry Analysis

**Figure 1:**
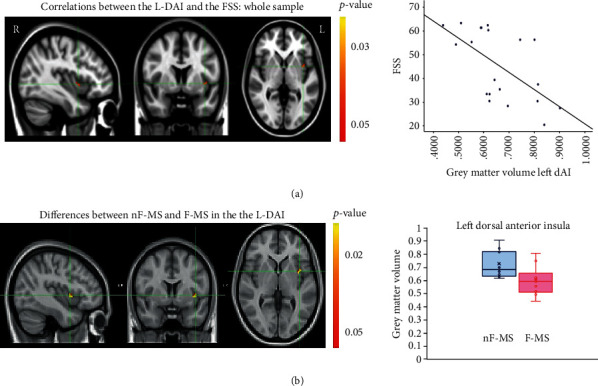
(a) Correlations between the dorsal anterior insular cortex (L-DAI) and the level of fatigue (FSS). In the *y*-axis, the different scores in the FSS are presented (higher scores mean more fatigue). In the *x*-axis, different values of GM volume in the L-DAI are presented. (b) Significant differences between the nF-MS and F-MS groups in the L-DAI. In the *y*-axis are represented the GM volume. In the *x*-axis, there are represented the two groups (nF-MS and F-MS).

The analysis with VBM comparing the nF-MS with the F-MS groups for the six ROI collapsed showed that the F-MS group had a significantly reduced GM in the left dAI when compared to the nF-MS group [voxels = 19, *p* = 0.018, peak coordinates in MNI (64, 65, 36)] (Table 2, Figure 1(b)).

### 3.4. Volumetric Analysis from the Insular Cortex

A mixed 2 by 2 ANOVA, between subject factor GROUP (F-MS versus nF-MS), within subject factor IC VOLUME (right IC versus left IC) showed that there was a significant volume difference between the right IC and the left IC (main effect of IC, *F*_(1, 18)_ = 50.911, *p* = 0.000, *η*_p_^2^ = 0.739, large size effect). The left IC was smaller (0.55% of TBV) than the right IC (0.60% of TBV). No significant interactions between groups and IC were found.

### 3.5. Correlations between Left dAI and the Whole GM Volume from the Insular Cortex and Fatigue

From the VBM analysis, considering the whole sample (*n* = 20), the left dAI showed a more reduced GM volume in individuals who reported higher FSS (rho = −0.647; *p* = 0.002, *R*^2^ = 0.369) (Figure 1(a)). There was no significant influence between the VAS_F and the GM volume. When considering the F-MS and the nF-MS groups independently, significant correlations were lost.

From the volumetric analysis, considering the whole sample (*n* = 20), negative correlations between the left and the right IC and the FSS scores were found. That is, the left and the right IC showed reduced GM volumes when the FSS scores were higher (rho = −0.470, *p* = 0.037, *R*^2^ = 0.221; rho = −0.473, *p* = 0.035, *R*^2^ = 0.224, respectively). No other significant correlations were found.

### 3.6. Anatomical Covariance Analysis (ACA)

**Figure 2:**
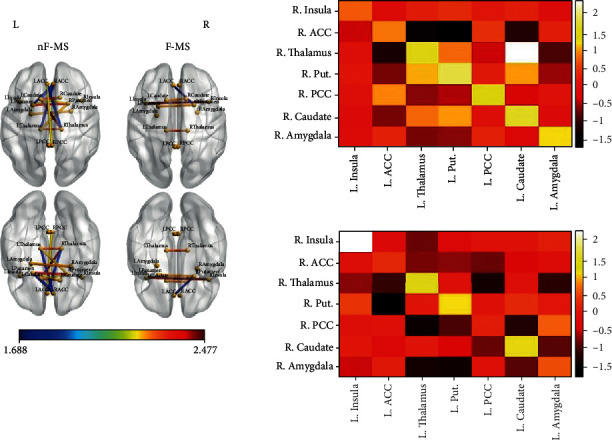
Results from the anatomical covariance analysis (ACA). The left column of glass brains belongs to the nF-MS group. The right column of brains belongs to the F-MS group. Blue lines indicate inverse correlations between the different volumes of interest (VOIs). Yellow and red indicate positive correlations between the VOIs. The color bar represents Fisher *Z* scores. Only significant correlations are presented. The right side of the figure shows the matrices of correlations between right and left VOIs. The up-right matrix belongs to the nF-MS group. The down-left matrix belongs to the F-MS group. L: left: R: right. The color bar of the matrices represents the Fisher *Z* scores.

Positive and negative significant bivariate Pearson correlations were found between the different pairs of VOIs in the nF-MS and the F-MS groups (Table 3, Figure 2). The F-MS group showed fewer significant correlations between the different anatomical volumes integrating the CoStN in comparison with the nF-MS group. The following correlations were no longer significant in the F-MS group in comparison with the nF-MS group: between the right ACC and the left ACC, thalamus, putamen, and caudate; between the right thalamus and the left ACC, between the right putamen and the left thalamus and caudate; between the right PCC and the left ACC and PCC; between the right caudate and the left thalamus and putamen. Additionally, new positive significant correlations between the right putamen and PCC and the left amygdala, and a new negative significant correlation appeared between the right PCC and the left thalamus.

## 4. Discussion

To our knowledge, this is the first study to assess [1] the role of the IC in self-reported trait and state fatigue in PwMS as a primary goal, and [2] how the interoceptive anatomical regions are related to different anatomical structures involved in cognitive, motor, and psychosocial fatigue. The results showed that as levels of trait fatigue increased, the left dAI showed a reduction in GM volume across the whole sample (*n* = 20). Once the sample was divided into nF-MS and F-MS groups, a significant GM reduction in left dAI was found in the F-MS group compared with the nF-MS group. This result supports our first hypothesis, and it is consistent with current literature [13, 14]. All three studies illustrate the need to include the IC in understanding the “feeling” of fatigue. The IC, as part of the Salience Network (SN), is a highly connected region mainly involved in the representation, interpretation, and integration of internal and external signals coordinating external and internal attentional processes [45]. It has been suggested [46] that when the functionality of the IC is disturbed, the level of vigilance and alertness towards external stimuli becomes compromised due to the interference of interoceptive stimuli [46]. The interference of interoceptive stimuli may, in turn, disturb the processing of external signals needed to perform a task [46] making challenging to focus the attention on external oriented tasks. Therefore, a higher effort is needed to complete and external oriented task. This higher effort, sustained along the time, could be the cause of fatigue in PwMS.

As hypothesized in our second hypothesis, no significant correlations were found between state fatigue and GM atrophy in the IC. State fatigue, considered as a dynamic process that fluctuates depending on external factors [11], has been mainly related to fMRI tasks in which attentional resources are required and sustained during a long period of time. Therefore, both the difficulty/cognitive load of the task and the time on task are key components to assess how state fatigue increases gradually along the time. Those two components can only be assessed with fMRI paradigms in which fluctuations of functional activity can be related to changes [12] in state fatigue.

Finally, as hypothesized in our third hypothesis, the group with clinical fatigue (F-MS) showed less GM correlations between different anatomical regions from the CoStN in comparison with the group without clinical fatigue (nF-MS). In our present work, the nF-MS group was characterized by [1] negative correlations between the left ACC and the right thalamus, putamen, and caudate; and between the left amygdala and the right thalamus, and [2] the preservation of the positive correlations between the right and the left homologous contralateral anatomical areas. Conversely, the F-MS group was mainly characterized by [1] the loss of correlations between the ACC and the rest of the anatomical regions considered in this study, [2] the emergence of two new positive correlations involving the amygdala (right putamen-left amygdala and right PCC-left amygdala). Whereas positive correlations can be suggestive of healthy GM volume between the connected structures, negative correlations are indicative of enough damage that need to be compensated [47]. Therefore, the presence of several negative correlations in the nF-MS group mainly involving the ACC might be suggestive of a process of compensation in areas involved in fatigue in PwMS before the development of clinical fatigue. Conversely, the loss of those same negative correlations involving the ACC in the F-MS group might be suggestive of the inability of the ACC to regulate fatigue, and the appearance of two new positive correlations involving the amygdala is suggestive of a change in the structural organization of the brain in which a dysfunctional ACC and an atrophied IC might be being replaced by other anatomical structures such as the amygdala. The ACC, the IC, and the amygdala, as interoceptive brain areas, have been recently related to fatigue in PwMS (Campo et al., 2019; Hanken et al., 2018).

This study has some limitations. First, there was a small sample size. Secondly, it was cross-sectional in nature. Third, there was a lack of an appropriate matched healthy control group, and a lack of measures of physical disability, pharmacological therapy, and depression, which limits the application of the findings to an extended population, prevents the establishment of causality and restricts further interpretations of the different pattern of correlations found in both groups.

In conclusion, we found that GM atrophy in the IC is related to higher levels of trait fatigue but not with state fatigue, and changes in the IC, the ACC and the amygdala are involved in the process of fatigue in PwMS. This implies that trait fatigue is a more complex system that can be better understood when considering the interoceptive component in its study. Furthermore, future studies focused on the study of fatigue should include not only measures of fatigue but also measures of interoceptive perception to complement the assessment of fatigue. Most importantly, interoceptive training could be a therapeutic approximation to alleviate fatigue in PwMS.

Given the small sample and the pilot nature of the study, the lack of significant correlations should not be interpreted as evidence of a lack of a relationship. Therefore, the results reported should be interpreted with caution, specially the lack of correlation between the IC and state fatigue. Future longitudinal studies with larger samples are warranted to understand the neural changes associated with fatigue not only through the disease but also through the different types of the disease.

## Figures and Tables

**Table 1:** Demographic and clinical information of the sample.

Variable	nF-MS group (*n* = 10)	F-MS group (*n* = 10)	*p* value
	M (SD)	M (SD)	
Age	45.5 (10.9)	49.5 (8.8)	n.s.
Gender/male, *n* (%)	2 (20%)	1 (10%)	n.s.
Education	15.3 (1.8)	16.3 (2.6)	n.s.
Disease duration (months)	118.8 (56.1)	198.1 (122.3)	n.s.
MS type, *n* (%)			
RRMS	5 (62.5%)	7 (77.8%)	
PPMS	1 (12.5%)	0	n.s.
SPMS	2 (25%)	1 (11.1%)	
PRMS	0	1 (11.1%)	
FSS	31.2 (5.5)	59.0 (3.37)	<0.005
VAS_F	8.7 (9.8)	26.5 (25.2)	0.059
SDMT, raw scores	51.5 (16.2)	50.8 (11.6)	n.s.

MS: multiple sclerosis; RRMS: Relapsing-Remitting MS; PPMS: Primary Progressive MS; SPMS: Secondary Progressive MS; PPRMS: Progressive Primary Relapsing MS; FSS: Fatigue Severity Scale; VAS_F: Visual Analogue Scale for Fatigue; SDMT: Symbol Digit Modality Test; M: mean; SD: (standard deviation); *n*: number. See [22] for the MS type diagnosis criteria.

**Table 2:** Decreased areas of GM volume in the whole group and in the F-MS group. Values are FWE corrected.

Anatomical region	Cluster size	Hemisphere	*p* value	MNI coordinates		
Whole sample						
Dorsal anterior IC	10	Left	0.027	64	65	36
nF_MS > F-MS						
Dorsal anterior IC	19	Left	0.018	65	65	36

MNI: Montreal Neurological Institute.

**(a):** 

nF-MS group							
Left							
Right	IC	ACC	Thal.	Put.	PCC	Caud.	Amyg.
IC	0.66 (0.04)	n.s.	n.s.	n.s.	n.s.	n.s.	n.s.
ACC	n.s.	0.74 (0.02)	-0.88 (0.00)	-0.93 (0.00)	n.s.	-0.78 (0.01)	n.s.
Thal.	n.s.	-0.81 (0.01)	0.92 (0.00)	0.71 (0.02)	n.s.	0.99 (0.03)	-0.67 (0.04)
Put.	n.s.	n.s.	0.82 (0.00)	0.96 (0.00)	n.s.	0.78 (0.00)	n.s.
PCC	n.s.	0.76 (0.01)	n.s.	n.s.	0.91 (0.00)	n.s.	n.s.
Caud.	n.s.	n.s.	0.72 (0.02)	0.79 (0.01)	n.s.	0.94 (0.00)	n.s.
Amyg.	n.s.	n.s.	n.s.	n.s.	n.s.	n.s.	0.85 (0.00)

**(b):** 

F-MS group							
Left							
Right	IC	ACC	Thal.	Put.	PCC	Caud.	Amyg.
IC	0.98 (0.00)	n.s.	n.s.	n.s.	n.s.	n.s.	n.s.
ACC	n.s.	n.s.	n.s.	n.s.	n.s.	n.s.	n.s.
Thal.	n.s.	n.s.	0.91 (0.00)	n.s.	n.s.	n.s.	n.s.
Put.	n.s.	n.s.	n.s.	0.86 (0.00)	n.s.	n.s.	0.77 (0.00)
PCC	n.s.	n.s.	-0.64 (0.05)	n.s.	n.s.	n.s.	0.72 (0.02)
Caud.	n.s.	n.s.	n.s.	n.s.	n.s.	0.88 (0.00)	n.s.
Amyg.	n.s.	n.s.	n.s.	n.s.	n.s.	n.s.	0.71 (0.01)

IC: insular cortex; ACC: anterior cingulate cortex; Thal.: thalamus; Put.: putamen; PCC: posterior cingulate cortex; Caud.: caudate; Amyg.: amygdala. The first number refers to the correlation's coefficient; the second number between parentheses refers to the significance of the correlation.

## Data Availability

De-identified data will be shared with any qualified investigator by request.
